# Construction of nomogram for the clinical outcome for pediatric acute lymphoblastic leukemia patients

**DOI:** 10.1097/MD.0000000000050259

**Published:** 2026-08-28

**Authors:** Yutong Sang, Ruixiang Li, Yiwen Gao, Wenhua Zhang, Jiahua Hu

**Affiliations:** a Department of Pediatrics, Affiliated Hospital of Shandong Second Medical University, Weifang, China.

**Keywords:** acute lymphoblastic leukemia, nomogram, prognosis factor

## Abstract

Acute lymphoblastic leukemia (ALL) is one of the most common hematologic cancers in children, accounting for about 25% of all pediatric cancers. However, survival data for pediatric ALL patients remain limited. We selected clinicopathological data from pediatric ALL patients diagnosed between 2000 and 2019 from the SEER database. Univariable and multivariable Cox proportional hazards regression analyses were applied to identify independent prognostic factors for overall survival (OS) in ALL patients. A predictive nomogram was then constructed. Calibration curves, receiver operating characteristic (ROC) curves, and decision curve analysis (DCA) were used to evaluate the nomogram’s performance. A total of 13,869 ALL patients were included in the study, with 1447 (10%) patients dying from various causes. On univariable analysis, survival was significantly worse in male patients, those aged 13–17, and Black patients. Multivariable analysis identified age, race, and radiotherapy as independent prognostic factors for OS, whereas sex was not independently associated with survival. The nomogram predicted the 1-year, 3-year, and 5-year OS rates with AUCs of 0.807, 0.796, and 0.772 in the training cohort, and 0.854, 0.837, and 0.816 in the validation cohort, respectively. Good agreement was observed between observed outcomes and predicted probabilities in the calibration curves. DCA further demonstrated the nomogram’s clinical utility. The nomogram demonstrates good accuracy and reliability in predicting OS for pediatric ALL patients, providing valuable theoretical support for clinicians in making treatment decisions.

## 1. Introduction

Acute lymphoblastic leukemia (ALL) is one of the most common pediatric hematologic cancers, accounting for approximately 25% of all pediatric cancers.^[1]^ ALL can be categorized into T-cell ALL, B-cell ALL, and mixed-phenotype acute leukemia based on immunophenotype.^[2]^ Over the past few decades, significant advances in the treatment of childhood and adolescent ALL patients have been made, particularly with the advent of immunotherapies such as chimeric antigen receptor T-cell (CAR-T) therapy.^[3]^ Despite these advances, the survival rate for relapsed patients remains suboptimal. Therefore, there is an urgent need to develop models that predict clinical outcomes in pediatric ALL patients, which could better guide treatment stratification and management.

In this study, we aimed to explore the demographic characteristics and independent prognostic factors for pediatric ALL patients. A nomogram was developed to predict the OS rate using data from the SEER database. This study could offer a reference for the clinical identification of high-risk patients and assist in individualized treatment decisions.

## 2. Methods

### 2.1. Data source

The data for ALL cases were obtained from the SEER 17 Population-Based Registries Research Plus Data (2000–2019), which covers approximately 34.6% of the U.S. population. The SEER*Stat v8.4.3 software was used to collect and process the data for patients diagnosed with ALL. Only cases with complete information on age, sex, race, radiotherapy, chemotherapy, median household income, and follow-up were included in the study.

### 2.2. Study variables

The baseline characteristics of all patients included age, sex, race, median household income, radiotherapy, chemotherapy, time from diagnosis to treatment, survival status, and survival time. Only pediatric patients, defined as those ages 0–17 years at the time of diagnosis, were included in the study. Patients were further categorized by race into white, Black, and other groups. Median household income was divided into 2 categories: <$75,000 and ≥$75,000. The primary outcome (event of interest) was death from any cause, and patients who remained alive were censored at the last follow-up. Because the endpoint was all-cause mortality, there was no competing event in this analysis, and the SEER cause-specific death classification was therefore not used. Accordingly, the nomogram was constructed to predict overall survival rather than ALL-specific mortality. Survival time was defined as the time from ALL diagnosis to either the date of death or December 30, 2020.

### 2.3. Statistical analysis

Univariable and multivariable Cox proportional hazards regression models were employed to identify independent prognostic factors for overall survival, and variables significant in univariable analysis were entered into the multivariable model. Nomograms were constructed using the “rms” and “survival” packages in R software. The performance of the nomograms was evaluated using the Harrell concordance index (C-index) and ROC curves. Statistical analyses were performed with SPSS 25.0 software (IBM Corp., USA) and R 4.1.1 software (R Core Team, Austria). All tests were two-sided, and *P* < .05 was considered statistically significant.

## 3. Results

### 3.1. Clinical characteristics of ALL patients

**Figure 1.**
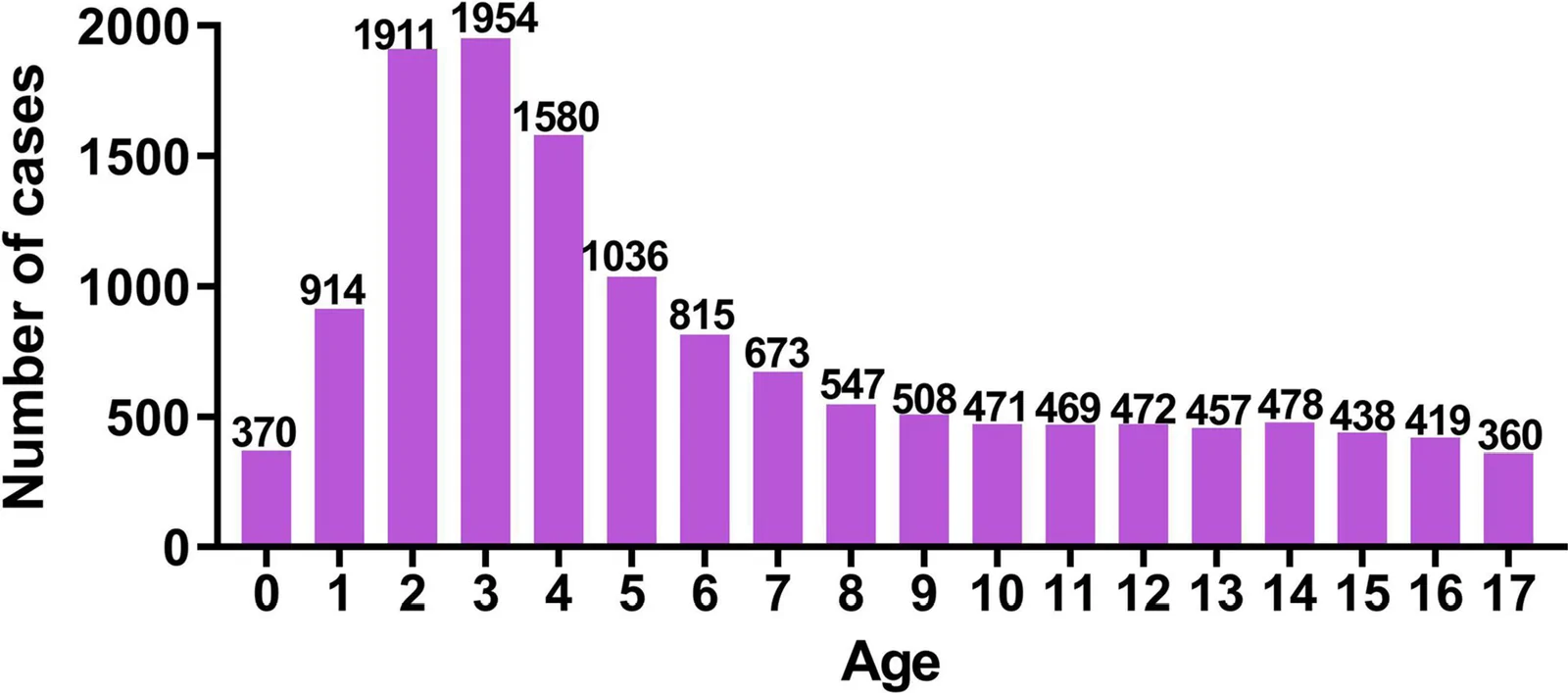
Age distribution dichotomized of acute lymphoblastic leukemia patients. The age distribution is based on the age at diagnosis, with the sample size (N) indicated for each age group.

As shown in Table 1, a total of 13,869 ALL patients from the SEER database were included in the study, with a random division into two cohorts (7:3 ratio): the training cohort (n = 9708) and the validation cohort (n = 4161). Most of the patients (83%) were of white race. Among these, 7799 (56%) patients were male. During the follow-up period, 1447 (10%) patients died from various causes. Figure 1 shows the age distribution of the study cohort, with the majority of patients diagnosed at ages 2–4. Detailed patient characteristics are provided in Table 1.

**Table 1:** Baseline characteristics of the training and validation cohorts.

	All cohort	Training cohort	Validation cohort	*P*-value
	N = 13869 (100%)	N = 9708 (70%)	N = 4161(30%)	
**Age** *	5 (3, 10)	5 (3, 10)	5 (3, 10)	.858
**Race**				.812
White	11,520 (83%)	8022 (83%)	3498 (84%)	
Black	992 (7.2%)	681 (7.0%)	311 (7.5%)	
Other	1357 (9.8%)	1005 (10%)	352 (8.5%)	
**Sex**				.725
Female	6070 (44%)	4223 (44%)	1847 (44%)	
Male	7799 (56%)	5485 (56%)	2314 (56%)	
**Radiotherapy**				.831
No	12569 (90.6%)	8805 (90.7%)	3674 (90.5%)	
Yes	1300 (9.4%)	903 (9.3%)	397 (9.5%)	
**Chemotherapy**				.781
No	44 (0.1%)	31 (0.1%)	13 (0.1%)	
Yes	13825 (99.9%)	9677 (99.9%)	4148 (99.9%)	
** Income**				.898
$75,000+	4318 (31%)	3015 (31%)	1303 (31%)	
< $75,000	9551 (69%)	6693 (69%)	2858 (69%)	
** Survival status**				.894
Alive	12,422 (90%)	8669 (89%)	3753 (90%)	
Dead	1447 (10%)	1039 (11%)	408 (9.8%)	
**Time**	7.7 (3.3, 13.6)	7.6 (3.3, 13.5)	7.8 (3.3, 13.8)	

* Age is represented as the median (interquartile range).

### 3.2. Survival and risk factors for ALL patients

**Figure 2.**
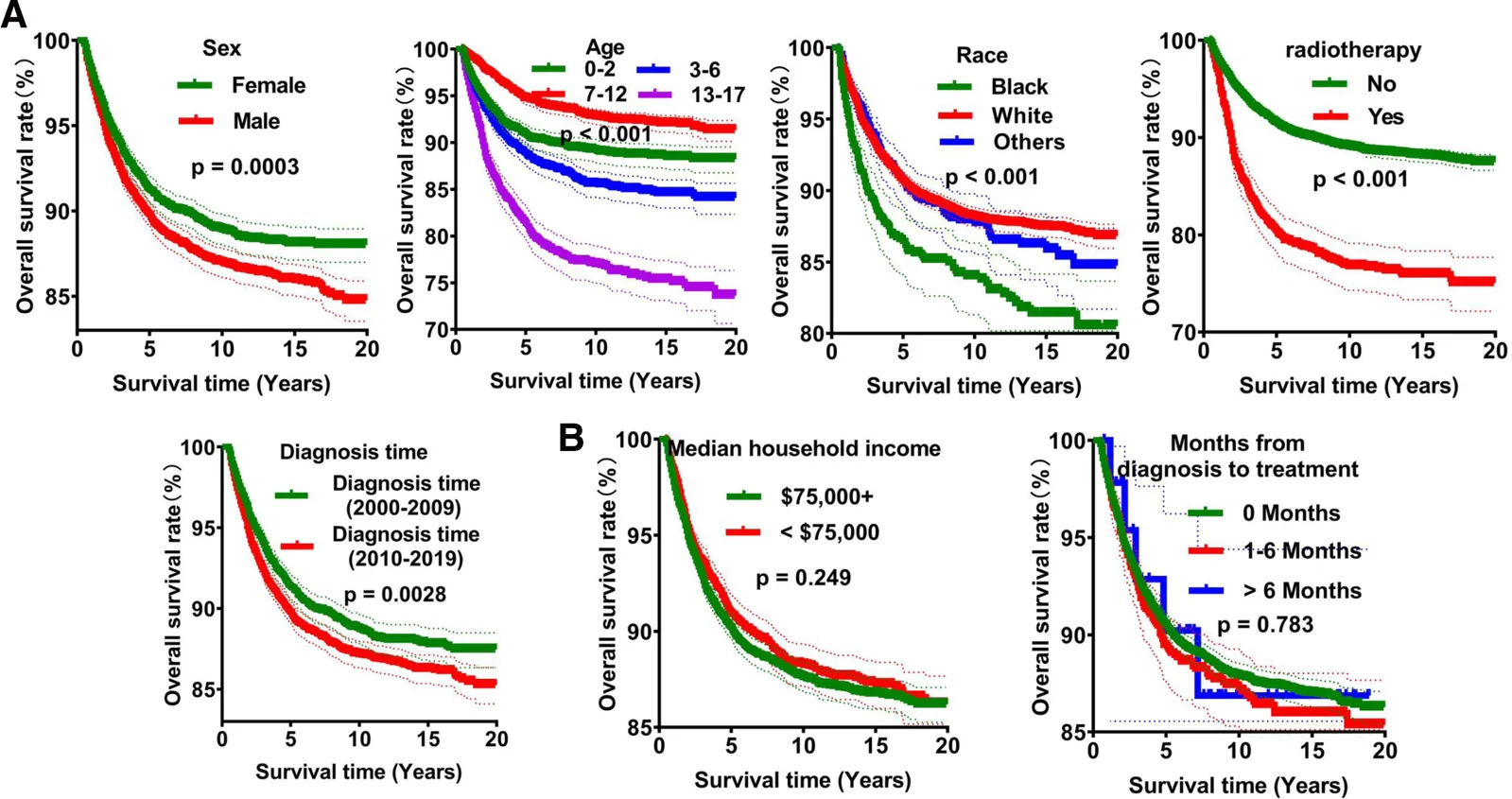
The clinical outcome of acute lymphoblastic leukemia patients. The overall survival curve in acute lymphoblastic leukemia patients in different (A) sex, age, race, radiotherapy status, diagnosis time, and (B) median household income, months from diagnosis to treatments. The dotted lines represent the confidence intervals for the survival estimates, showing the variability around the main survival curve.

Male ALL patients had a worse overall survival (OS) rate compared to female patients (Fig. 2A, *P* = .003). Among the different age groups, ALL patients aged 7–12 had the best clinical outcomes, while patients aged 13–17 had the worst (Fig. 2A, *P* < .001). Compared with Black patients, White and other race patients had better OS rates (Fig. 2A, *P* < .001). Survival was significantly worse in ALL patients treated with radiotherapy (Fig. 2A, *P* < .001). Interestingly, patients diagnosed between 2010 and 2019 had better OS compared to those diagnosed from 2000 to 2009 (Fig. 2A, *P* = .0028), reflecting improvements in treatment and supportive care. No significant differences in OS were observed based on median household income, time from diagnosis to treatment, or chemotherapy status (Fig. 2B, all *P* > .05). Univariable analysis identified age, race, sex, and radiotherapy as factors associated with OS. In the multivariable model, age, race, and radiotherapy remained independent prognostic factors: patients of “Other” race had a significantly higher risk of death than White patients (HR 1.41, 95% CI 1.19–1.68, *P* < .001), whereas the association for Black race was attenuated and no longer statistically significant (HR 1.08, 95% CI 0.91–1.29, *P* = .376). Male sex showed only a borderline association with OS (HR 1.10, 95% CI 0.99–1.23, *P* = .067) and therefore did not meet the prespecified *P* < .05 criterion for an independent predictor (Table 2).

**Table 2:** Univariable and multivariable Cox proportional hazards regression analyses of factors associated with overall survival.

	Univariate analysis		Multivariate analysis	
	HR (95% CI)	*P*-value	HR (95% CI)	*P*-value
**Age**				
** **0–2	Ref.		Ref.	
** **3–6	0.61 (0.52–0.72)	< .001	0.61 (0.52–0.72)	< .001
** **7–12	1.31 (1.13–1.52)	< .001	1.21 (1.04–1.41)	.013
** **13–17	2.27 (1.96–2.63)	< .001	2.00 (1.72–2.32)	< .001
**Race**				
** **White	Ref.		Ref.	
** **Black	1.07 (0.89–1.27)	.479	1.08 (0.91–1.29)	.376
** **Other	1.53 (1.29–1.81)	< .001	1.41 (1.19–1.68)	< .001
**Sex**				
** **Female	Ref.		Ref.	
** **Male	1.21 (1.09–1.35)	< .001	1.10 (0.99–1.23)	.067
**Median household income**				
** **$75,000+	Ref.			
** **< $75,000	1.07 (0.95–1.20)	.249		
**Months from diagnosis to treatment**				
** **0	**Ref.**			
** **1–6	1.08 (0.91–1.28)	.377		
** **7+	0.99 (0.41–2.37)	.975		
**Radiotherapy**				
** **No	Ref.		Ref.	
** **Yes	2.28 (2.00–2.59)	< .001	1.72 (1.50–1.96)	<.001
**Chemotherapy**				
** **No	Ref.			
** **Yes	0.94 (0.35–2.50)	.894		

CI = Confidence interval, HR = Hazard ratio.

The HR represents the hazard ratio for death from any cause in the Cox proportional hazards model, comparing each category to the reference group.

### 3.3. Establishment of the prediction nomogram

**Figure 3.**
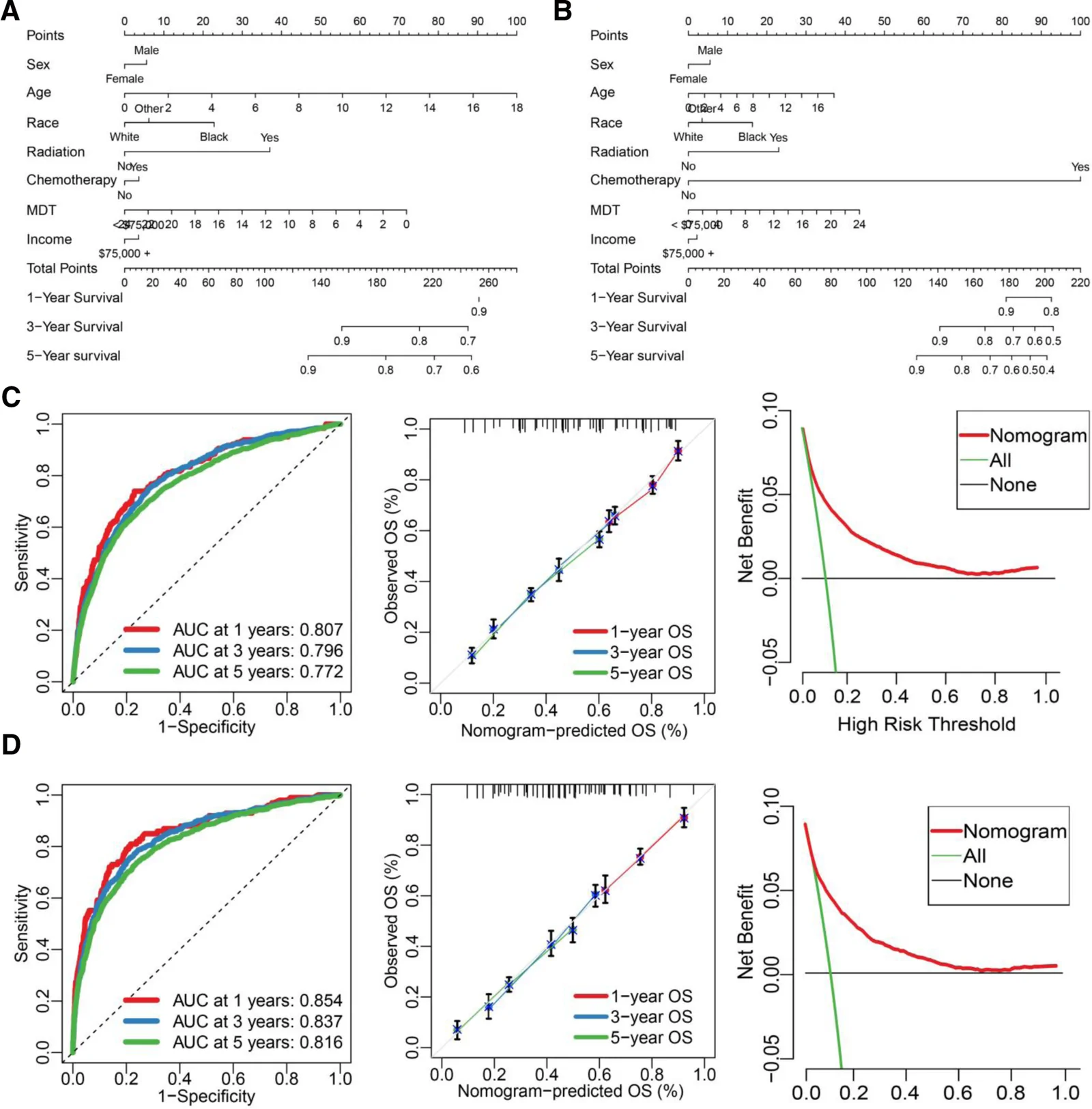
Construction and evaluation of the predicting nomogram. (A, B) The nomogram for predicting the overall survival of acute lymphoblastic leukemia patients in the training and testing cohorts. The top row of the “Points” represents a scale for each risk factors, and points of each predictor were acquired by drawing a straight line upwards from the corresponding value to the “Points” line. Then, the points received from each predictor are summed, and the number is located on the “Total Points” axis. To conclude the patient’s sort of probability for overall survival at certain year, draw a straight line down to the corresponding “X-Year Survival” axis. Receiver operating characteristic curve, calibration curve and decision curve analysis curve for evaluating the performance of nomogram in training (C) and testing (D) cohort.

We constructed a nomogram for predicting OS in the training cohort and validated it in the validation cohort (Fig. 3A, B). The total score for each patient was calculated by summing the points for each variable, which predicts the probability of death from any cause (i.e., the 1-, 3-, and 5-year overall survival probabilities).

### 3.4. Evaluation of the performance of the prediction nomogram

**Figure 4.**
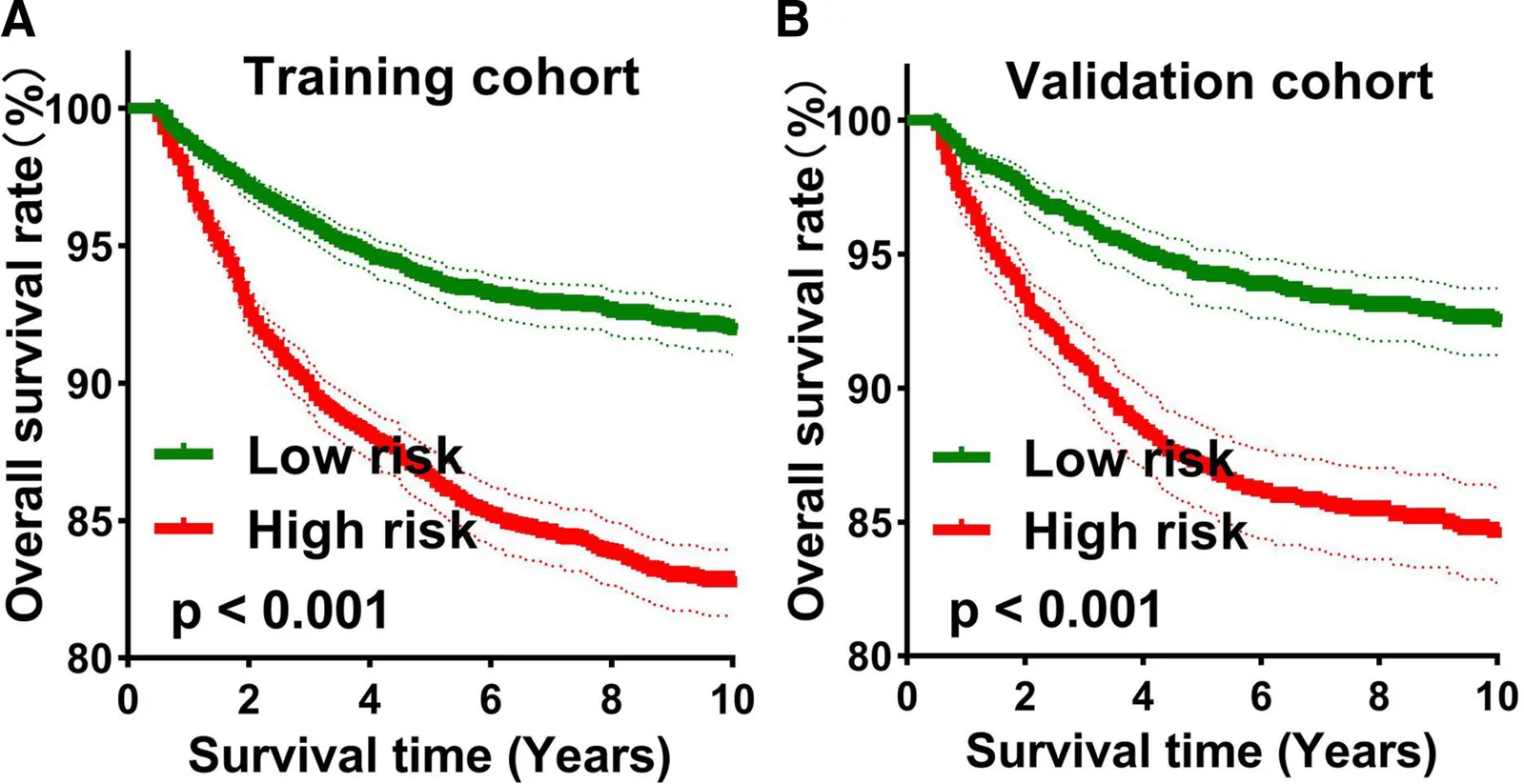
The clinical outcome of acute lymphoblastic leukemia in different groups. The clinical outcome of acute lymphoblastic leukemia with different risk scores in the training (A) and validation (B) cohorts. The dotted lines represent the confidence intervals for the survival estimates, showing the variability around the main survival curve.

ROC curves for the prediction nomogram indicated the following AUCs for the 1-year, 3-year, and 5-year OS rates: 0.807, 0.796, and 0.772 in the training cohort, and 0.854, 0.837, and 0.816 in the validation cohort, respectively (Fig. 3C, D). Additionally, good agreement was observed between observed outcomes and predicted probabilities in the calibration curves for both the training and validation cohorts (Fig. 3C, D). Further DCA demonstrated the good clinical utility of the nomogram models (Fig. 3C, D). The cohort was divided into high and low-risk groups based on risk score, with high-risk patients showing worse OS in both the training and validation cohorts (Fig. 4A, B).

## 4. Discussion

In this study, we identified independent risk factors for the OS rate of pediatric ALL patients and constructed a predictive nomogram that demonstrates good accuracy and reliability. This nomogram provides valuable support for clinicians to make informed decisions about treatment. When cancer-specific mortality is the endpoint of interest, deaths from other causes act as competing events, and conventional survival analysis may overestimate the risk of cancer-specific mortality because it does not account for competing risks.^[4]^ Austin et al introduced the competing risk framework for analyzing survival data in the presence of competing risks.^[5]^ Consistent with this, cancer-specific mortality estimated by traditional survival analysis methods has been shown to be higher than that estimated by competing risk models in settings where competing events are present.^[6]^ In the present study, however, the primary endpoint was overall survival, with death from any cause as the event of interest; consequently, there were no competing events, and univariable and multivariable Cox proportional hazards regression were used to identify independent prognostic factors. Using this approach, we identified age, race, and radiotherapy as independent prognostic factors for the clinical outcome of pediatric ALL patients.

Our study showed that age is a risk factor for the OS rate of pediatric ALL patients, and older age within the pediatric population correlated with a poor OS rate. Regarding age and prognosis, numerous studies have shown that younger pediatric ALL patients tend to have more favorable outcomes, though there are exceptions depending on other clinical factors. Age is a well-established prognostic factor for ALL, with younger patients (typically those aged 1–9 years) often having a better overall survival (OS) rate compared to older patients, particularly those aged 10 years and above. This is likely due to differences in the biology of the disease and how it responds to treatment at different stages of development. For example, a study by Testi et al found that children with ALL who were younger than 10 years old had better event-free survival compared to older children and adolescents, suggesting that age-related biological factors play a role in treatment response.^[7]^ Similarly, studies such as those by Hunger et al have highlighted that children diagnosed at a younger age tend to have a more favorable prognosis due to a combination of better treatment response and lower incidence of treatment-resistant disease.^[8]^ However, the risk of relapse and poor outcomes tends to increase as patients get older, which has been consistently reported in both cohort studies and clinical trials. Our findings align with these observations, as we found that pediatric patients aged 7–12 had the best clinical outcomes, while those aged 13–17 had the worst, reinforcing the significance of age as a prognostic factor in pediatric ALL.

Regarding race and sex, univariable analysis suggested worse OS in male and Black patients (Fig. 2A); however, these associations were attenuated after multivariable adjustment. Male sex did not remain an independent predictor of OS (HR 1.10, 95% CI 0.99–1.23, *P* = .067), and Black race was not independently associated with mortality (HR 1.08, 95% CI 0.91–1.29, *P* = .376), suggesting that the univariable associations may be partly confounded by other clinical and demographic factors. In contrast, patients of “Other” race had a significantly higher risk of death than White patients (HR 1.41, 95% CI 1.19–1.68, *P* < .001), a finding that warrants further investigation. These results underscore the importance of multivariable adjustment when interpreting demographic prognostic factors.

There are several limitations in our study. This is a retrospective study, and therefore, there may be bias in case selection. Most patients received chemotherapy, but no details of which chemotherapeutic agents they received were available. The recent approval of novel generation TKIs, antibody-drug conjugates, and bispecific antibody constructs may not be fully reflected in the survival improvement in this study. It is possible that novel agents may further improve ALL outcomes with longer follow-up. Moreover, the data analyzed in this study came from cases in the United States, and the diagnosis and treatment of ALL in other countries is somewhat different from that in the United States, which also affects the extensibility of the model. Moreover, the number of patients who did not receive chemotherapy was very low (44 of 13,869 patients, 0.3%), which limited the statistical power to evaluate the prognostic effect of chemotherapy and may affect the accuracy of the nomogram. The lack of data on specific chemotherapy regimens, including newer agents like blinatumumab and allogeneic stem cell transplantation, is a limitation of this study. These factors may affect treatment outcomes and should be considered in future research.

## 5. Conclusion

The nomogram demonstrates good accuracy and reliability in predicting OS for pediatric ALL patients, providing valuable theoretical support for clinicians in making treatment decisions.

## Author contributions

**Methodology:** Yutong Sang, Ruixiang Li.

**Supervision:** Yutong Sang.

**Validation:** Yutong Sang, Ruixiang Li.

**Writing – review & editing:** Yutong Sang, Ruixiang Li, Yiwen Gao, Jiahua Hu.

**Formal analysis:** Yiwen Gao, Jiahua Hu.

**Resources:** Yiwen Gao.

**Investigation:** Wenhua Zhang.

**Writing – original draft:** Wenhua Zhang.

**Project administration:** Jiahua Hu.
